# Diagnosis and Treatment of Hereditary Central Diabetes Insipidus in a Swiss Family With a Mutation in the *AVP* Gene

**DOI:** 10.1210/jcemcr/luac023

**Published:** 2022-12-03

**Authors:** Lorena Wyniger, Nicole Beuret, Jonas Rutishauser, Eleonora Seelig

**Affiliations:** Department of Endocrinology, Diabetology and Metabolism, University Hospital Basel, 4031 Basel, Switzerland; Biozentrum, University of Basel, 4056 Basel, Switzerland; Department of Endocrinology, Diabetology and Metabolism, University Hospital Basel, 4031 Basel, Switzerland; Department of Endocrinology, Diabetology and Metabolism, University Hospital Basel, 4031 Basel, Switzerland; University Clinic of Medicine, Cantonal Hospital Baselland, 4410 Liestal, Switzerland

**Keywords:** hereditary central diabetes insipidus, *AVP*, mutation, copeptin, *AVP* deficiency

## Abstract

Hereditary central diabetes insipidus (CDI) is a genetic disorder characterized by polydipsia and polyuria. Most known mutations are located in the arginine-vasopressin (*AVP*) gene. Here, we describe a Swiss family with an autosomal dominant mutation in the *AVP* gene region encoding for the carrier protein neurophysin II (P55R). In addition, we discuss the algorithm for diagnosing and treating patients with hereditary CDI based on this Swiss family.

Central diabetes insipidus (CDI) is characterized by polydipsia and hypotonic polyuria due to inadequate secretion or deficiency of the hormone arginine-vasopressin (AVP) from the hypothalamus or posterior pituitary. On changes in plasma osmolality and blood pressure, AVP is released in the circulation and binds to AVP type 2 receptors in the collecting ducts, where it promotes free water reabsorption [1].

CDI is mainly acquired by disorders inflicting damage to the hypothalamus or pituitary. In some cases, however, CDI is caused by genetic mutations. Most of these mutations are in the *AVP* gene and cause a classical polyuria-polydipsia phenotype [2]. The diagnosis of hereditary CDI is elaborate but crucial because of impending risks such as hypernatremia.

Here we describe the phenotype and genotype of a Swiss family with a mutation in the *AVP* gene region encoding for the carrier protein neurophysin II (P55R). The family history of polyuria and polydipsia gives insight into the medical challenges of hereditary CDI. We discuss the algorithm for diagnosing patients with hereditary CDI based on the Swiss family. We describe how the use of copeptin and genetic testing can reduce the diagnostic burden. Furthermore, we discuss our treatment approach to patients with hereditary DI.

## Case Presentation

The index patient, a 28-year-old white woman, was referred by her general practitioner for an endocrine evaluation of polydipsia and polyuria.

The patient drank around 10 liters of fluids per day and produced large amounts of urine regularly. She had developed these symptoms during her early childhood.

Until recently, the patient had not been especially bothered by these symptoms. However, at the time of referral, she was going through a stressful period in her life. She felt that her nocturia harmed her sleep and quality of life, which eventually caused her to seek medical attention.

Apart from polydipsia and polyuria, her past medical history was unremarkable. She had no history of urinary tract infections, renal failure, or hypernatremia.

**Figure 1.**
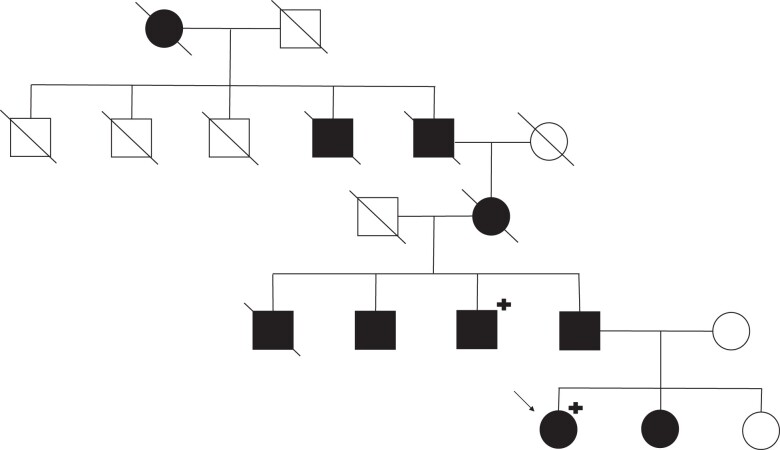
Family pedigree of hereditary central diabetes insipidus with autosomal dominant inheritance pattern. The black symbols indicate symptomatic individuals. The arrow indicates the index patient. + indicates genetically tested individuals. All diseased family members reached adulthood. The cause of death of the diseased family members is unknown.

Her family history revealed that several family members (dating back to the 19th century) were noted to drink excessive amounts of fluids. According to the family chronicle, her great-grandfather had a medical assessment for polydipsia in the 1950s. He was diagnosed with a “harmless endocrine disorder” and received some powder without treatment effect. In total, 9 additional family members had polyuria and polydipsia, including the patient's father and her sister (Fig. 1).

## Diagnostic Assessment

Physical examination and routine blood counts were regular. The glomerular filtration rate was normal (121 mL/min/1.73 m^2^, reference range > 90 mL/min/1.73 m^2^). Anterior pituitary hormones were intact. Serum sodium was 142 mmol/L (136-146 mmol/). Urine osmolality was 65 mOsmol/kg, and plasma osmolality was 296 mOsmol/kg. Baseline plasma copeptin was very low (0.92 pmol/L; reference range, 1.2-16.4 pmol/L); nephrogenic diabetes insipidus was therefore excluded. Stimulation with hypertonic saline infusion was performed to differentiate between CDI and primary polydipsia [3]. Copeptin did not significantly increase after stimulation (from < 1.23 pmol/L to 1.25 pmol/L, at plasma sodium 152 mmol/L), confirming the diagnosis of CDI. A magnetic resonance imaging scan showed no structural abnormality, especially no adenoma and no displacement or widening of the pituitary stalk. A hyperintense signal in the posterior pituitary was visible in T1-weighted images.

Genetic testing of the index patient revealed a C to G transition at nucleotide position 164, in 1 allele, resulting in the substitution of proline by arginine on position 24 (P55R) in exon 2 encoding neurophysin II. For genetic testing genomic DNA was isolated from blood/EDTA using the GenElute Mammalian Genomic DNA kit (Sigma). Exon 1 was amplified by polymerase chain reaction (PCR) using Q5 polymerase (NEB) with primers sense: TGCCTGAATCACTGCTGACCGCTGGGGACC and antisense: AACTTCCCCTAAAGGCTACC. Exon 2 + 3 was amplified by PCR using an Expand High Fidelity PCR System (NEB) with primers sense: GCCTGCAGCCGCAGCCCCGGTGTCCCGC and antisense: CCTCTCTCCCCTTCCCTCTTCCCGCCAGAG. PCR products were purified using a NucleoSpin Plasmid kit (Macherey Nagel) and sequenced by Microsynth. PCR and sequencing were repeated to confirm the presence of the mutation. Genetic testing was performed at the Biozentrum, University Basel.

The second case we examined was the paternal uncle, aged 52 years. The patient was drinking around 6 liters per day. Like his niece, he got up several times to drink and urinate at night. His symptoms started around age 7 years. Until age 35, he was drinking around 10 liters per day; the fluid volume has decreased since. His medical history includes obesity (body mass index 34.4), well-controlled diabetes mellitus type 2, and chronic venous disease. His glomerular filtration rate was normal (107 mL/min/1.73 m^2^). He reported no urinary tract infections or hypernatremia. The uncle was not bothered by polyuria and polydipsia. Genetic analysis confirmed the same mutation, P55R in exon 2 of the *AVP* gene.

## Treatment and Follow-up

The uncle was so accustomed to polyuria-polydipsia that he did not consider any therapy. In the young index patient, treatment with a modest bedtime dose of oral desmopressin (synthetic AVP) was initiated. After the initiation of therapy, the patient was frequently monitored for her response and to avoid hyponatremia. Treatment with desmopressin quickly resulted in marked regression of symptoms and improved quality of life.

## Discussion

Here we describe the genetic and clinical features of a family with hereditary CDI. The underlying cause is an autosomal dominant missense mutation in the *AVP* gene region encoding for the carrier protein neurophysin II (P55R).

Mutations in the same nucleotide position have been previously described in families with polyuria-polydipsia phenotype [4, 5]. In these families, proline was substituted with leucine (P55L) or histidine (P55H) rather than arginine as in the Swiss family. Overall, more than 75 mutations in the *AVP* gene causing hereditary CDI are known [2]. The *AVP* gene encodes a preproprotein consisting of AVP and 2 other proteins, namely the carrier protein neurophysin II and copeptin. All 3 proteins are packaged into neurosecretory vesicles and transported from the hypothalamus to the neurohypophysis for storage [2]. On changes in plasma osmolality and blood pressure, they are collectively released into the circulation. While AVP is essential for fluid homeostasis, the biological activity of neurophysin II and copeptin after secretion in the blood circulation is still unclear [1]. All known disease-causing *AVP* mutations are either located in the gene region encoding *AVP* or neurophysin II and typically result in a polyuria-polydipsia phenotype. Very rarely, CDI is caused by mutations in *WFS1* or *PCSK1*, and these patients usually develop additional symptoms such as diabetes mellitus [2].

Mutations in the *AVP* gene are generally transmitted in an autosomal dominant fashion (> 90%), and only a few families show a recessive inheritance pattern. In the Swiss family, the autosomal dominant inheritance pattern is evident as the mutation is present in every generation and equally affects males and females. Distinguishing the mode of inheritance is essential because it affects the onset of symptoms. In patients with rare autosomal recessive transmission, symptoms manifest acutely in the neonatal period with the danger of severe, life-threatening dehydration. With autosomal dominant inheritance, as in the Swiss family, symptoms typically develop later in childhood and progress gradually to severe polyuria [2]. The reason for this delayed onset of symptoms is under discussion. Preclinical data indicate that the mutant AVP precursor hormone does not pass the secretory protein control process, is retained in the endoplasmic reticulum, and is degraded by the cytosolic proteasome. Residual functional proteins produced by the nonaffected allele initially compensate for the loss of function of mutant proteins. However, pathological protein structures accumulate with time and impair functional proteins, leading to symptom onset [2, 6].

Although CDI is considered a progressive disease, symptoms occasionally improve in adulthood, like in the paternal uncle, who reduced his fluid intake with older age from around 10 to 6 liters per day. However, the underlying reason for this phenomenon is not apparent [2].

The diagnosis of DI is based on medical history, clinical examination, and laboratory testing. First, hypotonic polyuria (urinary volumes of > 50 mL/kg/day) and polydipsia (fluid intake > 3 L/day) need to be confirmed. Next, CDI has to be differentiated from nephrogenic DI and primary polydipsia [1]. Nephrogenic DI results from renal insensitivity to AVP; primary polydipsia describes the consumption of large fluid volumes and hypotonic polyuria with intact AVP activity. All 3 disorders present with similar symptoms, so laboratory testing, including stimulation tests, is needed to differentiate them [1].

**Figure 2.**
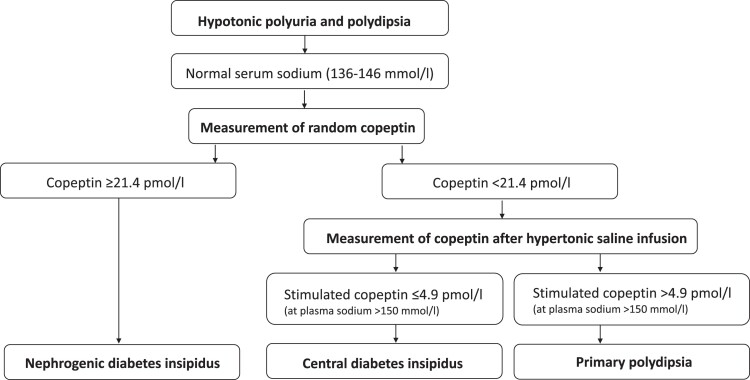
Copeptin-based algorithm for differential diagnosis of polyuria-polydipsia syndrome.

Until recently, the indirect water deprivation test was considered the gold standard for differentiating the cause of polyuria-polydipsia syndrome. However, this test has been validated in only a few patients; its diagnostic accuracy is low and cumbersome for patients [7]. Newer studies show that copeptin is a handy diagnostic tool to differentiate between CDI, nephrogenic DI, and primary polydipsia (Fig. 2) [3, 8]. Copeptin can be used as a surrogate marker for AVP as it is cosecreted in the circulation in an equimolar ratio to AVP. The advantage over AVP is that copeptin is stable and reliably measured with commercially available assays, whereas measuring AVP with commercially available assays is more error prone [7]. In nephrogenic DI, copeptin levels are high (≥ 21.4 pmol/L) [9]. If baseline copeptin levels are low, CDI can be differentiated from polydipsia with a hypertonic saline infusion or an arginine stimulation test [8]. Indeed, both tests have higher diagnostic accuracy than the commonly used water deprivation test [3]. The diagnostic accuracy and tolerability of the hypertonic saline infusion test and the arginine stimulation test are currently compared in a randomized controlled multicenter study (ClinicalTrials.gov NCT03572166). Here we used the hypertonic saline infusion test with a copeptin cutoff of 4.9 mol/L. In our index patient, baseline copeptin levels were very low (< 2 pmol/L) and did not increase with hypertonic saline. Based on these results, we could diagnose CDI without conducting a lengthy and cumbersome water deprivation test.

After the diagnosis of CDI is established, the next step generally is to exclude acquired forms such as tumors or trauma. Physical examination, medical history, and radiographic findings are helpful in this process. We next conducted a gadolinium-enhanced MRI of the hypothalamus and the pituitary to identify potential anatomical pathologies. The MRI scan was completely normal and showed an area of hyperintensity in the posterior pituitary in T1-weighted images, also referred to as a bright spot. The bright spot reflects high phospholipid concentrations found in the axonal membrane, the membrane of vesicles, and glial cells in the posterior pituitary [10]. The bright spot is typically visible in healthy individuals but often disappears in CDI [1]. Our patient with long-lasting DI but visible bright spot on the MRI scan confirms previous findings indicating a limited diagnostic accuracy of the bright spot in CDI [3].

Due to the family history of several generations with polydipsia-polyuria syndrome, we next initiated genetic studies. Genetic analysis is generally advised if there is a positive family history or a childhood manifestation of symptoms [1, 2]. Furthermore, genetic testing is beneficial as detecting a genetic mutation in a family can reduce the burden of further strenuous diagnostic testing for relatives, which is especially important in children.

Since many individuals with hereditary CDI are not diagnosed until later in life, the question of the urgency of the diagnosis arises. Indeed, the disease can be managed relatively well as long as patients can self-regulate their fluid intake. However, undiagnosed DI can quickly become critical if patients are incapacitated and can no longer drink in response to perceived thirst. Operations, old age, immobility, and cognitive impairment are risk factors for developing severe dehydration with hypernatremia in undiagnosed patients with DI. Furthermore, untreated chronic polyuria can dilate the ureters and bladders, causing vesicoureteral reflux, ascending urinary tract infections, and secondary renal insufficiency [2]. Therefore, timely diagnosis is essential.

Next to avoiding life-threatening situations, the main treatment goal in CDI is to improve quality of life. Pharmacological therapy with oral or nasal desmopressin is readily available and well tolerated [1]. We usually initiate therapy with a modest bedtime dose of desmopressin to reduce nocturia. A single dose of desmopressin often does not suffice to control symptoms for 24 hours. We add a second dose during the daytime depending on the recurrence of symptoms and the patient's well-being. We generally aim for a minimum dose of desmopressin as treatment can result in hyponatremia. Initial monitoring of sodium levels and patient information are essential to avoid hyponatremia. Hyponatremia occurs if patients on desmopressin habitually continue drinking ample amounts of fluids.

In summary, we have discovered a mutation in this family with hereditary CDI. This mutation is described as P55R and is located in the *AVP* gene's NPII region (exon 2). For diagnosis and treatment of hereditary CDI, a step-wise approach based on copeptin measurement is recommended. Genetic testing is advised if a genetic background is suspected or known, as it can alleviate the diagnostic burden and drive therapy.

## Learning Points

- Hereditary CDI is caused mainly by autosomal dominant mutations in the *AVP* gene, with a polyuria-polydipsia phenotype developing during childhood.
- A copeptin-based diagnostic algorithm facilitates the diagnosis of CDI.
- Genetic testing is advised if family history is suspicious and symptoms develop during childhood.
- Treatment with desmopressin improves quality of life and reduces the risk of urinary tract infections and renal insufficiency.

## Data Availability

Restrictions apply to the availability of some data to preserve patient confidentiality. The corresponding author will on request detail the restrictions and any conditions under which access to some data may be provided.

## Abbreviations

*AVP*: arginine-vasopressin
CDI: central diabetes insipidus
DI: diabetes insipidus
MRI: magnetic resonance imaging
P55R: protein neurophysin II
PCR: polymerase chain reaction
